# Supplementary material for: Statistical integration of multi-omics and drug screening data from cell lines
Source: PLoS Comput Biol. 2024 Jan 31;20(1):e1011809. doi: 10.1371/journal.pcbi.1011809 (PMC10878536; doi:10.1371/journal.pcbi.1011809)
Supplement: S1 Supporting information — The mathematical details and proofs for POPLS-DA are given, as well as additional results and figures for the data analysis. (PDF) [file pcbi.1011809.s001.pdf]

Supplement to "Statistical integration of multi-omics and drug screening data from cell lines"

Said el Bouhaddani[1,*†], Matthias Höllerhage[2†], Hae-Won Uh[1], Claudia Moebius[3], Marc Bickle[4], Günter Höglinger[2,5,6‡], Jeanine Houwing-Duistermaat[1,7‡]

1 Dept. Data science & Biostatistics, UMC Utrecht, Utrecht, Netherlands
2 Department of Neurology, Hannover Medical School, Hannover, Germany
3 Roche Institute for Translational Bioengineering, Basel, Switzerland
4 Max Planck Institute of Molecular Cell Biology and Genetics, Dresden, Germany
5 Department of Neurology, Ludwig-Maximilians-Universität, Munich, Germany
6 German Center for Neurodegenerative Diseases, Munich, Germany
7 Dept. of Mathematics, Radboud University, Nijmegen, Netherlands

†These authors contributed equally to this work.
‡These authors also contributed equally to this work.
* sbouhad2@umcutrecht.nl

1 Introduction

This document is a supplement to the main article "Statistical integration of multi-omics data identifies potential therapeutic targets for MSA". It is structured into two parts.

The first part, Section 2, contains the mathematical details of POPLS-DA and corresponding proofs. Also, the maximum likelihood estimation algorithm is presented. We discuss a strategy to select the number of joint components and the number of relevant genes. We additionally discuss how to interpret the POPLS-DA parameters.

The second part contains additional tables and figures for the multi-omics and bioinformatics analyses. We show scree plots for selecting the number of joint components and number of relevant genes. We perform a permutation test to assess whether the top 200 genes can significantly distinguish the two experimental groups. A table of these relevant genes is presented, as well as the GO enrichment clusters for those genes that were targeted by a drug.

2 Methods

For a joint analysis of the transcriptomics and proteomics datasets, we propose probabilistic orthogonal partial least squares discriminant analysis (POPLS-DA).

2.1 Mathematical model of POPLS-DA

As in the main article, x_t and x_p are random vectors of size d representing the transcriptomics and proteomics data. Further, y_t and y_p are univariate random variables representing dummy variables for the experimental conditions (one for cells overexpressing αSyn, and zero for the controls). In the POPLS-DA model, the omics data and the experimental conditions are linked via latent variables u_t and u_p of size r, where r is much smaller than the data dimensions. Specific components v_t and v_p are added to the model to capture variation of the omics data that doesn't play a role in

discriminating the two experimental groups. The numbers of specific components are r_t and r_p, respectively. Residual variation is modeled by including noise terms e_t and e_p for the transcriptomics and proteomics datasets, and ϵ_t and ϵ_p for the random variable representing the two conditions. The joint and specific latent variables u_t, u_p, v_t and v_p are assumed to be standard normally distributed. The residual terms e_t, e_p, ϵ_t and ϵ_p are normally distributed with zero mean and (co)variance (matrix) $\sigma^2_{e_t} I_d$, $\sigma^2_{e_p} I_d$, $\sigma^2_{\epsilon_t}$, and $\sigma^2_{\epsilon_p}$.

The mathematical model can be written as

$$\begin{aligned} x_t &= u_t W^{\mathrm{T}} + v_t P_t^{\mathrm{T}} + e_t & \quad x_p &= u_p W^{\mathrm{T}} + v_p P_p^{\mathrm{T}} + e_p, \\ y_t &= u_t \beta + \epsilon_t & \quad y_p &= u_p \beta + \epsilon_p. \end{aligned} \tag{1}$$

The matrices W of size $d \times r$, P_t of size $d \times r_t$ and P_p of size $d \times r_p$ contain the joint and specific loadings for each gene. The vector β contains the regression coefficients of y_t and y_p on the joint components u_t and u_p.

In the POPLS-DA model, (x_t, y_t) is normally distributed with zero mean and covariance matrix

$$\Sigma_t = \begin{bmatrix} WW^{\mathrm{T}} + P_t P_t^{\mathrm{T}} + \sigma^2_{e_t} I_d & W\beta \\ \beta W^{\mathrm{T}} & \beta^{\mathrm{T}}\beta + \sigma^2_{\epsilon_t} \end{bmatrix}. \tag{2}$$

Analogously, (x_p, y_p) is normally distributed with zero mean and covariance matrix denoted by Σ_p. The set of all parameters is denoted by $\theta = \{W, \beta, P_t, P_p, \sigma_{e_t}, \sigma_{e_p}, \sigma_{\epsilon_t}, \sigma_{\epsilon_p}\}$.

2.2 Estimation with maximum likelihood

The data from all samples are collected in X_t, X_p Y_t, and Y_p. Let $X = [X_t^{\mathrm{T}}, X_p^{\mathrm{T}}]^{\mathrm{T}}$ and $Y = [Y_t^{\mathrm{T}}, Y_p^{\mathrm{T}}]^{\mathrm{T}}$ be the datasets stacked across the rows. We propose to optimize the POPLS-DA likelihood by implementing an expectation-maximization (EM) algorithm. The log likelihood is given by

$$l(\theta|X,Y) = f((X_t, Y_t), \mu = 0, \Sigma = \Sigma_t) \cdot f((X_p, y_p), \mu = 0, \Sigma = \Sigma_p) \tag{3}$$

where f is a multivariate normal density function with the given mean and covariance matrix.

We propose to optimize the likelihood using EM, where the complete data likelihood can be written (with abuse of notation) as

$$l_c(\theta) = f(X_t|U_t, V_t) f(Y_t|U_t) f(U_t) f(V_t) \cdot f(X_p|U_p, V_p) f(Y_p|U_p) f(U_p) f(V_p). \tag{4}$$

In an EM algorithm, the conditional expectation of $l_c(\theta)$ given X and Y is optimized. Note that the optimizations can be decoupled and performed per term. For example, the first term yields the optimization problem,

$$\max_\theta \mathbb{E}[\log f(X_t|U_t, V_t)|X_t, Y_t] + \mathbb{E}[\log f(X_p|U_p, V_p)|X_p, Y_p]. \tag{5}$$

With respect to W, this becomes

$$\min_W \mathbb{E}[||X_t - U_t W^{\mathrm{T}} - V_t P_t^{\mathrm{T}}||_F^2 | X_t, Y_t] + \mathbb{E}[||X_p - U_p W^{\mathrm{T}} - V_p P_p^{\mathrm{T}}||_F^2 | X_p, Y_p]. \tag{6}$$

In the expectation step, we calculate the conditional expectations of the latent variables. We focus on the x_t terms. First, note that we can rewrite the POPLS-DA model as

$$(x_t, y_t) = (u_t, v_t)\Gamma^{\mathrm{T}} + (e_t, \epsilon_t), \tag{7}$$

with $\Gamma_t = \begin{pmatrix} W & P \\ \beta^{\mathrm{T}} & 0 \end{pmatrix}$. Using the rules for conditional expectations of normal distributions (e.g. see [1]) , we get

$$\begin{aligned} \mathbb{E}[(u_t, v_t)|x_t, y_t] &= (x_t, y_t)\Sigma^{-1}_{(e_s,\epsilon_s)}\Gamma\tilde{\Sigma}_{u_t,v_t}, \\ \mathrm{Var}[(u_t, v_t)|x_t, y_t] &= \tilde{\Sigma}_{u_t,v_t} \end{aligned} \tag{8}$$

where $\tilde{\Sigma}_{u_t,v_t} = (I - \Gamma^{\mathrm{T}}\Sigma^{-1}_{(e_s,\epsilon_s)}\Gamma)$. By replacing t with p, the conditional expectations given (x_p, y_p) are obtained. With these expressions, the objective function (6) can be optimized.

In the maximization step for W, we solve the optimization problem (6). Note that both terms depend on W. Taking the derivative with respect to W and equating to zero yields

$$\begin{aligned} X_t^{\mathrm{T}}\mathbb{E}[U_t|X_t, Y_t] - P_t\mathbb{E}[U_t^{\mathrm{T}}V_t|X_t, Y_t] + X_p^{\mathrm{T}}\mathbb{E}[U_p|X_p, Y_p] - P_p\mathbb{E}[U_p^{\mathrm{T}}V_p|X_p, Y_p] = \\ W\{\mathbb{E}[U_t^{\mathrm{T}}U_t|X_t, Y_t] + \mathbb{E}[U_p^{\mathrm{T}}U_p|X_p, Y_p]\} \end{aligned} \tag{9}$$

The expected values are taken from the expectation step. This yields the maimization step for W, which is given below. Optimizers for the other terms of the complete likelihood are obtained similarly. The complete EM algorithm for POPLS-DA is given by the following iterative scheme in k, starting with initial values for $k = 0$ and $s = t, p$,

$$\begin{aligned} W^{k+1} &= (\sum_{s=t,p} X_s^{\mathrm{T}}\mathbb{E}_k[U_s] - P_s^k\mathbb{E}_k[V_s]^{\mathrm{T}}\mathbb{E}_k[U_s])(\sum_{s=t,p}\mathbb{E}_k[U_s^{\mathrm{T}}U_s])^{-1} \\ \beta^{k+1} &= (\sum_{s=t,p} Y_s^{\mathrm{T}}\mathbb{E}_k[U_s])(\sum_{s=t,p}\mathbb{E}_k[U_s^{\mathrm{T}}U_s])^{-1} \\ P_s^{k+1} &= (X_s^{\mathrm{T}}\mathbb{E}_k[V_s] - W^{k+1}\mathbb{E}_k[U_s]^{\mathrm{T}}\mathbb{E}_k[V_s])(\mathbb{E}_k[V_s^{\mathrm{T}}V_s])^{-1} \\ (\sigma^2_{e_s})^{k+1} &= (N_s d)^{-1}\mathbb{E}_k[E_s^{\mathrm{T}}E_s] \\ (\sigma^2_{\epsilon_s})^{k+1} &= (N_s)^{-1}\mathbb{E}_k[\mathcal{E}_s^{\mathrm{T}}\mathcal{E}_s] \end{aligned} \tag{10}$$

In this scheme, $\mathbb{E}_k[\cdot] = \mathbb{E}[\cdot|X, Y, \theta^k]$.

2.3 Interpretation of POPLS-DA.

For sake of brevity, we will drop the subscript t and p in this paragraph. In the article, x in Equation (1) represents the transcriptomics and proteomics data. The experimental conditions for each omics dataset are indicated by y. POPLS-DA models the relationship between all omics data and conditions simultaneously in terms of the joint latent variables u. The loadings W represent the projection of the omics data onto the u. In our model, the loading weight for gene j in joint principal component k is given by $w_{j,k}$. A larger weight indicates a larger relative contribution to that joint component. Furthermore, if $w_{j,k}$ and $w_{j',k}$ have the same sign, the corresponding genes j and j' are positively correlated within component k. The number $(W\beta)_j = \sum_k w_{j,k}\beta_k$ is the coefficient indicating the relationship between gene j with the experimental groups. The vector $W\beta$ can be interpreted as the covariance of y with all omics data x. The latent variable $u_{i,k}$ indicates the position of data point x_i in the joint component k. Two subjects i and i' are similarly positioned if $u_{i,k}$ and $u_{i',k}$ are similar numbers. The interpretation of v and P goes analogously. A visual schedule of the estimation is given in Figure 1.

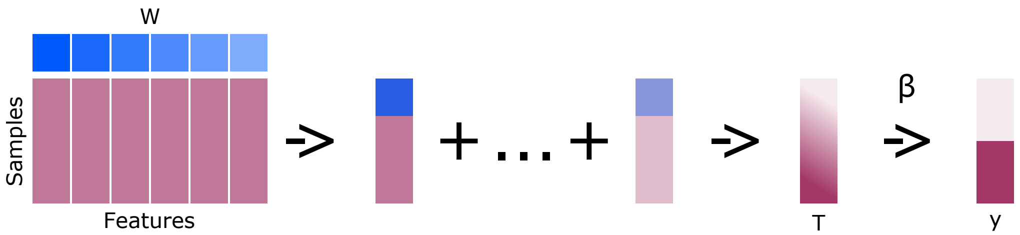

Figure 1. A visual scheme of the POPLS-DA component estimation. The features (purple rectangles) are given weights W (in blue) according to their relative importance in modeling the outcome y. The linear combination of features, weighted by the weights, form a component T. The weights are chosen such that the joint log-likelihood is optimized, where the distance between $T\beta$ and y is minimized.

2.4 Dimension selection.

The POPLS-DA model is formulated conditional on the number of joint and specific components, r and r_x, respectively. To estimate the number of components and the number of variables to investigate, we consider scree plots [2]. For the number of components, we calculate the eigenvalues of the stacked dataset $X = [X_1^{\mathrm{T}}, \ldots, X_s^{\mathrm{T}}]^{\mathrm{T}}$ and inspect the corresponding scree plot. To determine the number of variables per component to investigate, we calculate the estimated effect size $W\beta$ of each variable and use the "elbow" criterion on the sorted effect sizes (as in a scree plot [2]).

The proportions of variance explained by the joint and specific parts are calculated by dividing the trace of the corresponding covariance matrices by the trace of the modeled covariance matrix. In particular, the proportion of the variance of x_t explained by the joint part can be calculated by $\mathrm{tr}W^{\mathrm{T}}W$ divided by $\mathrm{tr}W^{\mathrm{T}}W + \mathrm{tr}P_t^{\mathrm{T}}P_t + d\sigma_{e_t}^2$.

3 Results

In this section, we present the supplementary figures and tables corresponding to the data analysis in the main article.

Scree plots of the eigenvalues of transcriptomics and proteomics datasets are shown in Figure 3, together with a scree plot of the effect sizes $W\beta$. Based on visually assessing where a plateau occurs, two joint and two specific components were selected. Furthermore, based on the visual assessment of the occurrence of an elbow, 200 genes were retained for interactome and functional analyses. These genes are given in Table 1.

We performed a permutation test where we randomly shuffled the case-control label for each sample. We then apply POPLS-DA again and calculate the overall accuracy (number of samples correctly classified based on the top 200 genes). The number of permutations was 400. In one permutation round, POPLS-DA achieved a perfect classification, corresponding with a p-value of 0.0025 (standard error: 0.0025). The median overall accuracy was 41.7% (equivalent to 10 out of 24 samples correct classifications).

The GO enrichment clusters for the subset of genes targeted by a drug are shown in Table 2. The list of drugs with their direct neighbors are shown in Table 3.

Finally, the String-DB networks from Fig. 3 are shown here enlarged in Figure 4, 5, 6, and 7.

Table 1. Top 200 genes in the joint transcriptomics-proteomics part. The weights are estimated with POPLS-DA, and indicate the contribution of each gene towards the classification.

weight	SYMBOL	weight	SYMBOL	weight	SYMBOL	weight	SYMBOL
0.338	MTHFD2	0.264	ESYT1	0.242	DDX3X	0.227	AATF
0.33	CBS	0.264	SLC4A7	0.242	MXRA7	0.227	ARSA
0.328	SCG2	0.264	PLD3	0.241	RPL11	0.227	UFD1L
0.326	TFRC	0.262	GPC6	0.24	PHGDH	0.227	MYO5A
0.322	NES	0.262	ALDH6A1	0.24	P2RX3	0.227	RP2
0.322	SLC3A2	0.262	MYCBP2	0.24	ARHGAP17	0.226	RPL6
0.322	SCARB2	0.262	RRM1	0.24	EEF2	0.226	WARS
0.322	FAT1	0.261	MAPK8IP3	0.239	SPTLC2	0.226	REM2
0.318	SLC7A5	0.261	SARM1	0.239	NEDD4L	0.226	ACBD3
0.318	POLDIP3	0.261	RCN2	0.239	TJP1	0.226	PDIA6
0.313	NRP2	0.261	PEG10	0.239	PON2	0.225	ENDOD1
0.312	ASNS	0.261	P4HB	0.238	HSPA5	0.225	SND1
0.312	AIMP2	0.261	SEC61A1	0.238	RPL38	0.225	NAV1
0.306	TNC	0.26	BCAT1	0.238	RPSA	0.225	PYCR1
0.306	CNTN2	0.259	ZYX	0.238	HYOU1	0.224	LAMP1
0.302	FABP3	0.258	DDX17	0.238	PSME2	0.224	TOR1B
0.301	RPS4X	0.258	GNS	0.237	MARS	0.224	SMPD1
0.301	SYT1	0.256	STX12	0.236	RRAS2	0.224	YBX1
0.298	STXBP1	0.256	ERGIC1	0.236	HSPB1	0.223	RAD21
0.297	SRL	0.256	ABCB6	0.235	ALDH9A1	0.223	CARS
0.297	PAFAH1B1	0.256	HPDL	0.235	VAV2	0.223	SEMA6D
0.296	PSAT1	0.255	CKMT1B	0.235	GAA	0.223	ADAR
0.296	KIF5C	0.254	NEFM	0.234	NUDT21	0.223	EEF1B2
0.294	BRD3	0.253	RPAP3	0.234	ARID3B	0.223	CSTF3
0.293	CNTNAP1	0.252	PRKAR2A	0.234	LUC7L2	0.223	SLC2A1
0.287	EEF1A2	0.251	ESD	0.234	KATNB1	0.223	POLR2A
0.285	OSBP	0.251	ENO3	0.234	ADD1	0.222	C11orf54
0.284	VIM	0.251	IQSEC1	0.234	CALU	0.222	RPS7
0.283	ACAT1	0.25	ATP6V1C1	0.234	VCL	0.222	HMGCR
0.283	IGF2R	0.25	GATAD2A	0.234	AP2A2	0.222	RPS3
0.282	SHMT2	0.249	VAT1	0.233	GLUD1	0.222	CLCN6
0.282	DNAJB11	0.249	USP9X	0.233	HDGFRP3	0.222	GLDC
0.282	NQO1	0.248	RPS24	0.233	YTHDC1	0.222	RPL4
0.281	RPL7A	0.248	SDF4	0.233	DICER1	0.221	EIF4B
0.28	CCDC50	0.248	YARS	0.232	RPS21	0.221	COL6A1
0.277	ECEL1	0.248	TAF15	0.232	CYB5A	0.221	RCC1
0.277	ALDH7A1	0.246	PDLIM3	0.232	GBA2	0.221	CADM4
0.277	GARS	0.246	ACTC1	0.231	NDUFA11	0.221	GOLM1
0.275	SPTBN1	0.246	FAR1	0.231	SMARCA1	0.221	AARS
0.275	MAP2K6	0.246	PDCD4	0.231	XPO5	0.22	THNSL1
0.27	MDGA1	0.245	HAX1	0.231	NCBP1	0.22	FAM171A1
0.269	RAB10	0.245	RAPGEF6	0.23	TARS	0.22	SLC7A6
0.267	RABGAP1	0.245	ANK2	0.23	MAP1A	0.22	CTSC
0.267	FYN	0.244	DSG2	0.23	MUT	0.22	SLC9A3R1
0.267	RCN1	0.244	RPL8	0.229	SYT11	0.22	SNRNP200
0.266	EPB41L5	0.244	SMC3	0.229	GRIPAP1	0.219	CLIC1
0.265	EIF5	0.243	OXCT1	0.229	APLP2	0.219	DPM1
0.265	LGALS3BP	0.242	FAHD2A	0.229	NAPB	0.219	HDGF
0.265	SLC1A4	0.242	FKBP2	0.229	NDUFA13	0.219	AKAP12
0.265	RFX3	0.242	ATP2C1	0.228	SPIN1	0.219	PSIP1

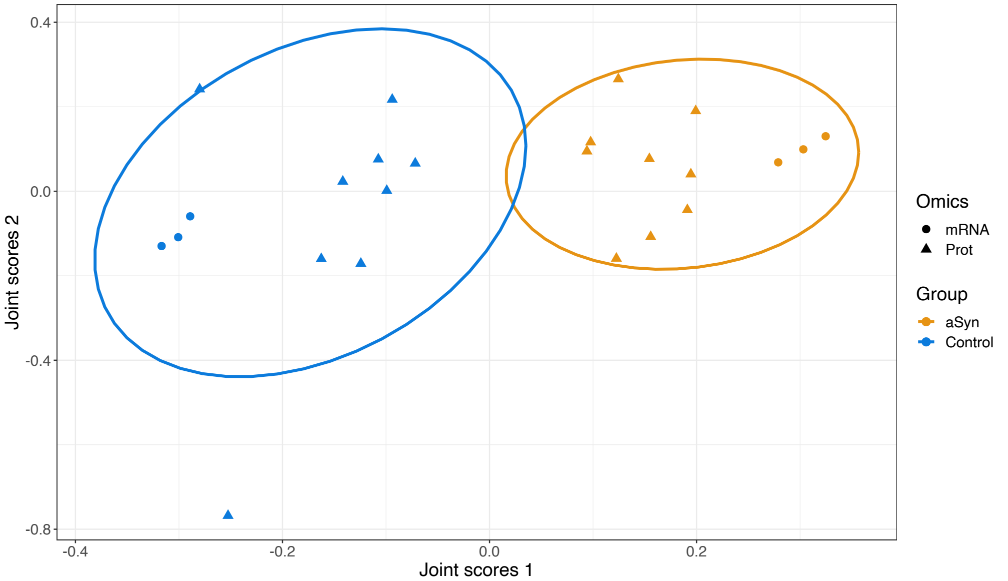

Figure 2. POPLS-DA joint scores. Each dot is an individual sample. Cell lines overexpressing α-synuclein are indicated by an yellow color, cell lines overexpressing the control protein are colored blue. A 90% confidence ellipse is added based on a t distribution.

Table 2. Gene ontology enrichment analysis of drug targeted genes. A subset of the top 200 genes, consisting of 116 genes that are (indirectly) targeted by an FDA approved drug compound, were used to perform GO enrichment analysis. The twenty most significant terms are shown.

Rank	Term	Ontology	Genes in set
1	extracellular exosome	CC	7.54e-22
2	extracellular vesicle	CC	7.78e-22
3	extracellular membrane-bounded organelle	CC	7.94e-22
4	extracellular organelle	CC	7.94e-22
5	cytoplasm	CC	2.39e-17
6	vesicle	CC	2.69e-16
7	extracellular space	CC	1.49e-15
8	cell junction	CC	1.05e-14
9	extracellular region	CC	3.19e-13
10	translation	BP	5.82e-12
11	amide biosynthetic process	BP	7.94e-12
12	peptide biosynthetic process	BP	1.33e-11
13	melanosome	CC	2.79e-11
14	pigment granule	CC	2.79e-11
15	RNA binding	MF	7.63e-11
16	cytoplasmic translation	BP	1.09e-10
17	developmental process	BP	1.33e-10
18	cellular amide metabolic process	BP	1.59e-10
19	focal adhesion	CC	2.08e-10
20	cell-substrate junction	CC	2.98e-10

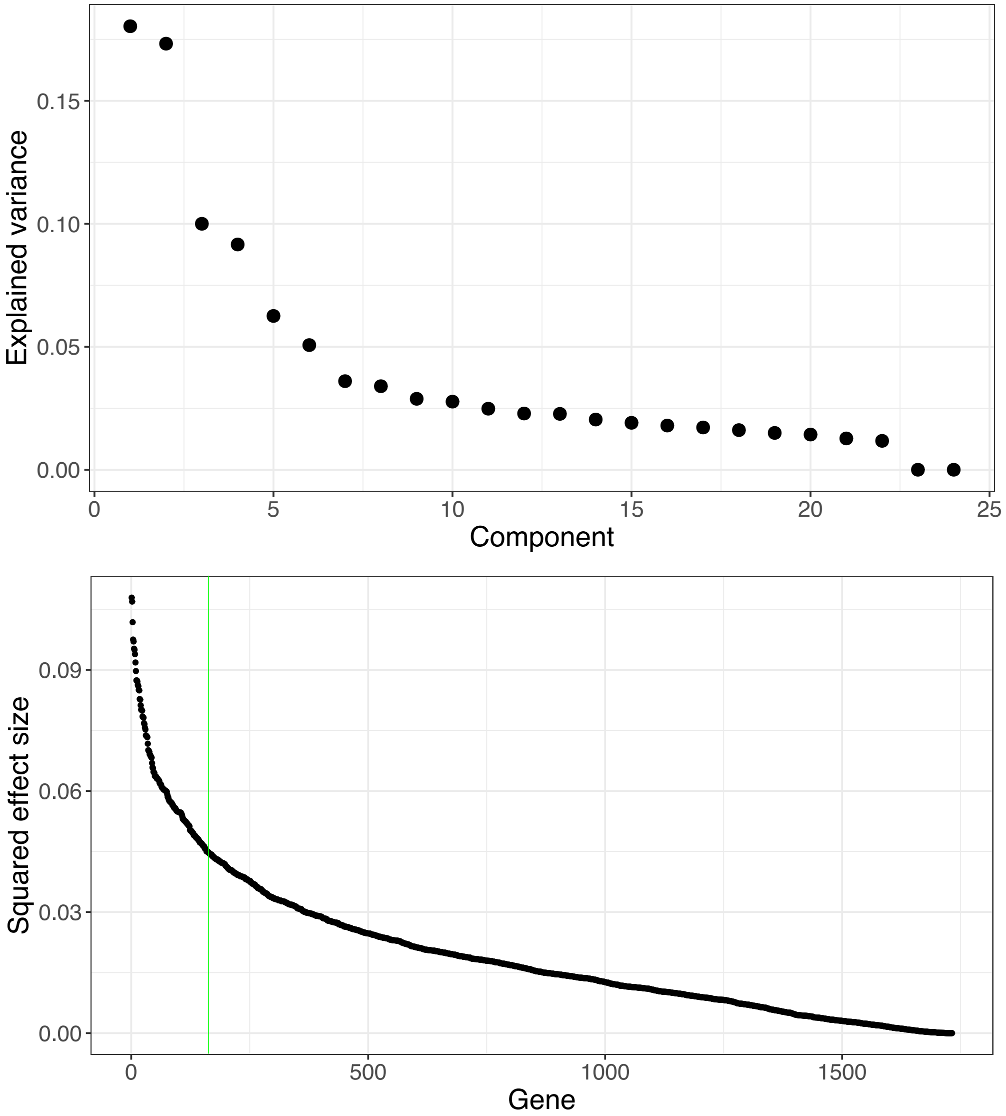

Figure 3. Scree plots for POPLS-DA. The number of components or genes is selected by visually assessing where a plateau or 'elbow' curve occurs. The *upper panel* shows the squared singular values of the stacked transcriptomics and proteomics data. The numbers are rescaled to sum up to one. The *lower panel* shows the sorted squared effect size per gene given by the squared elements of $W\beta$.

Table 3. List of the drugs and their direct neighbors. For each validated drug, the genes in the top 200 that interact with any drug target are shown.

Drug	Genes
Ajmaline	SPTBN1, ACTC1, ANK2, DSG2, SPTLC2, NEDD4L, NAV1
Amiodarone	CBS, SCG2, TFRC, NES, SCARB2, FAT1, TNC, FABP3, SYT1, EEF1A2, VIM, IGF2R, NQO1, FYN, RCN1, LGALS3BP, P4HB, GATAD2A, ACTC1, PDCD4, HAX1, EEF2, TJP1, PON2, HSPA5, RPSA, HSPB1, ALDH9A1, ADD1, CALU, DICER1, GBA2, NCBP1, APLP2, WARS, NAV1, PYCR1, LAMP1, YBX1, SLC2A1, HMGCR, CTSC, SLC9A3R1, CLIC1
Amlodipine	SCARB2, SYT1, STXBP1, NQO1, ESD, PDLIM3, ACTC1, HAX1, ANK2, SPTLC2, HSPA5, GAA, ADD1, GBA2, ARSA, REM2, NAV1, LAMP1, YBX1, SLC2A1, HMGCR
Astemizole	CBS, NES, CNTN2, STXBP1, KIF5C, NQO1, MDGA1, FYN, P4HB, NEFM, HAX1, ANK2, DSG2, EEF2, NEDD4L, HSPA5, ADD1, GBA2, NDUFA11, SYT11, APLP2, AATF, NAV1, YBX1, SLC2A1, HMGCR
Benazepril	MTHFD2, CBS, SLC3A2, SLC7A5, SHMT2, NQO1, SLC1A4, ABCB6, DSG2, MARS, GAA, ADD1, MUT, SLC2A1, HMGCR, CLCN6, GLDC, SLC7A6
Bepridil	POLDIP3, SYT1, RPS4X, STXBP1, VIM, IGF2R, NQO1, RCN2, PEG10, SEC61A1, ABCB6, ACTC1, HAX1, ANK2, DSG2, RPL8, DDX3X, RPL11, EEF2, NEDD4L, HSPA5, RPL38, ADD1, AP2A2, RPS21, GBA2, SYT11, MYO5A, REM2, NAV1, LAMP1, YBX1, SLC2A1, RPS7, HMGCR, RPS3
Pentoxyverine	ACAT1, ANK2, DSG2, P2RX3, NEDD4L, HSPA5, NAV1, YBX1, HMGCR, SLC9A3R1
Clemastine	NQO1
Dexibuprofen	CBS, SCG2, TFRC, NES, SLC3A2, SCARB2, FAT1, SLC7A5, TNC, FABP3, SYT1, EEF1A2, VIM, IGF2R, NQO1, FYN, RCN1, LGALS3BP, SLC1A4, SLC4A7, P4HB, SEC61A1, GNS, ABCB6, PRKAR2A, GATAD2A, VAT1, SDF4, ACTC1, PDCD4, HAX1, DSG2, RPL8, SMC3, EEF2, NEDD4L, TJP1, PON2, HSPA5, RPSA, PSME2, HSPB1, ADD1, CALU, AP2A2, DICER1, GBA2, SYT11, APLP2, WARS, PYCR1, LAMP1, YBX1, SLC2A1, HMGCR, RPS3, SLC7A6, CTSC, SLC9A3R1, CLIC1
Dicyclomine	TFRC, SLC3A2, SCARB2, SYT1, IGF2R, FKBP2, AP2A2, SYT11
Dipyridamole	DDX17, PRKAR2A, ESD, PDLIM3, HAX1, RPL11, SPTLC2, HSPA5, ALDH9A1, DICER1, GBA2, YBX1, SLC2A1, HMGCR, AARS, SLC9A3R1
Doxazosin	NQO1, HAX1, HSPA5, CALU, GBA2, YBX1, SLC2A1, HMGCR
Dyclonine	SPTBN1, ANK2, P2RX3, NAV1
Flunarizine	POLDIP3, SYT1, NQO1, RCN2, SEC61A1, ACTC1, ANK2, DDX3X, EEF2, ADD1, CALU, MYO5A, NAV1, HMGCR
Guanfacine	SLC7A5, IGF2R, NQO1, ALDH9A1, HMGCR
Ifenprodil	CNTN2, SYT1, STXBP1, SPTBN1, FYN, ANK2, APLP2
Imatinib	CBS, SCG2, TFRC, NES, NRP2, TNC, FABP3, SYT1, EEF1A2, VIM, IGF2R, NQO1, MAP2K6, FYN, RCN1, LGALS3BP, P4HB, ACTC1, HAX1, P2RX3, EEF2, SPTLC2, NEDD4L, TJP1, PON2, HSPA5, RPSA, HSPB1, ALDH9A1, VAV2, ADD1, CALU, AP2A2, DICER1, GBA2, APLP2, PYCR1, LAMP1, YBX1, SLC2A1, POLR2A, HMGCR, RPS3, CADM4, CTSC, SLC9A3R1, CLIC1
Lomerizine	NQO1, HAX1, HSPA5, GBA2, YBX1, SLC2A1, HMGCR
Nefazodone	SCG2, NQO1, NEFM, HAX1, ANK2, DSG2, NEDD4L, HSPA5, GBA2, MAP1A, NAV1, YBX1, CARS, SLC2A1, HMGCR
Pentamidine	SLC3A2, NQO1, YARS, ALDH9A1, ADD1, DICER1
Quinacrine	PAFAH1B1, VIM, IGF2R, NQO1, PLD3, HAX1, HSPA5, ALDH9A1, ADD1, GBA2, YBX1, SLC2A1, HMGCR
Reserpine	SCG2, SYT1, PAFAH1B1, IGF2R, EIF5, RRM1, USP9X, HAX1, SMC3, HSPA5, ALDH9A1, ADD1, GBA2, YBX1, RAD21, SLC2A1, HMGCR, SLC9A3R1

Continues on next page

Drug	Genes
Risperidone	CBS, SCG2, TFRC, NES, FAT1, TNC, FABP3, SYT1, EEF1A2, VIM, IGF2R, NQO1, FYN, RCN1, LGALS3BP, P4HB, NEFM, ACTC1, HAX1, EEF2, SPTLC2, TJP1, PON2, HSPA5, RPSA, HSPB1, ADD1, CALU, DICER1, GBA2, MAP1A, APLP2, PYCR1, LAMP1, YBX1, CARS, SLC2A1, HMGCR, CTSC, SLC9A3R1, CLIC1
Telmisartan	TFRC, NES, SCARB2, FABP3, SYT1, IGF2R, NQO1, GATAD2A, PDCD4, HAX1, TJP1, HSPA5, RPL38, ADD1, AP2A2, DICER1, GBA2, SYT11, WARS, SND1, YBX1, SLC2A1, HMGCR, SLC9A3R1
Triflupromazine	TFRC, SLC3A2, SCARB2, SYT1, IGF2R, HAX1, FKBP2, PON2, HSPA5, RRAS2, AP2A2, GBA2, SYT11, YBX1, SLC2A1, HMGCR
Trimipramine	SCG2, FAT1, NQO1, GARS, NEFM, HAX1, P2RX3, HSPA5, CALU, GBA2, MAP1A, YBX1, CARS, SLC2A1, HMGCR
Tropisetron	FAT1, GARS, P2RX3

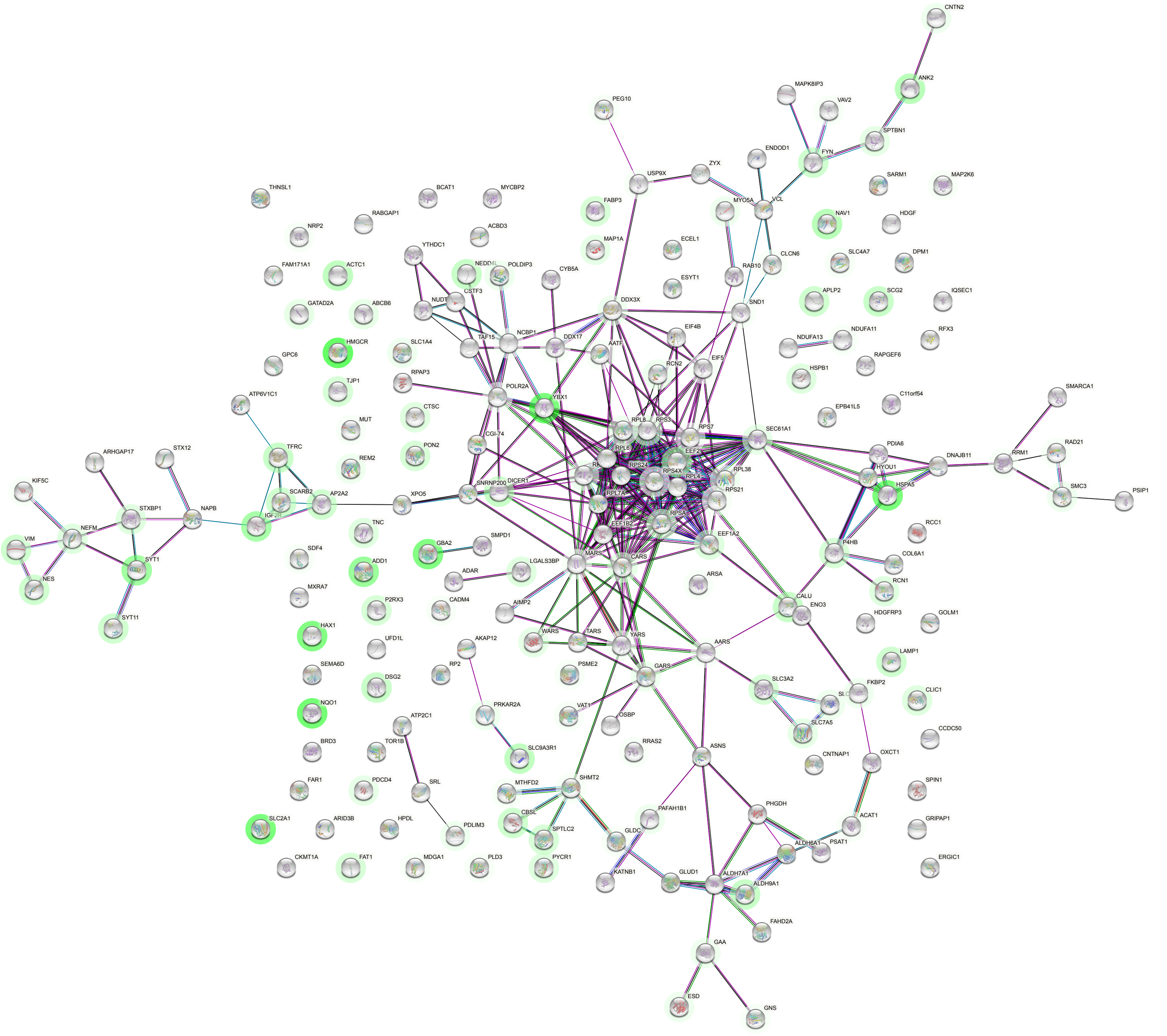

Figure 4. String-DB and clustering analyses of the top 200 genes/proteins. A network of interactions between the top 200 genes/proteins (estimated with POPLS-DA) was constructed using String-DB. Each node is a gene, and a connection between genes indicates evidence for a biologically plausible link. Text mining was excluded as an evidence source, and a medium confidence threshold was used. For genes that were (indirectly) targeted by a drug compound, a green 'halo' is drawn. The intensity of the green color is proportional to the number of drug compounds for which the gene was an (indirect) target.

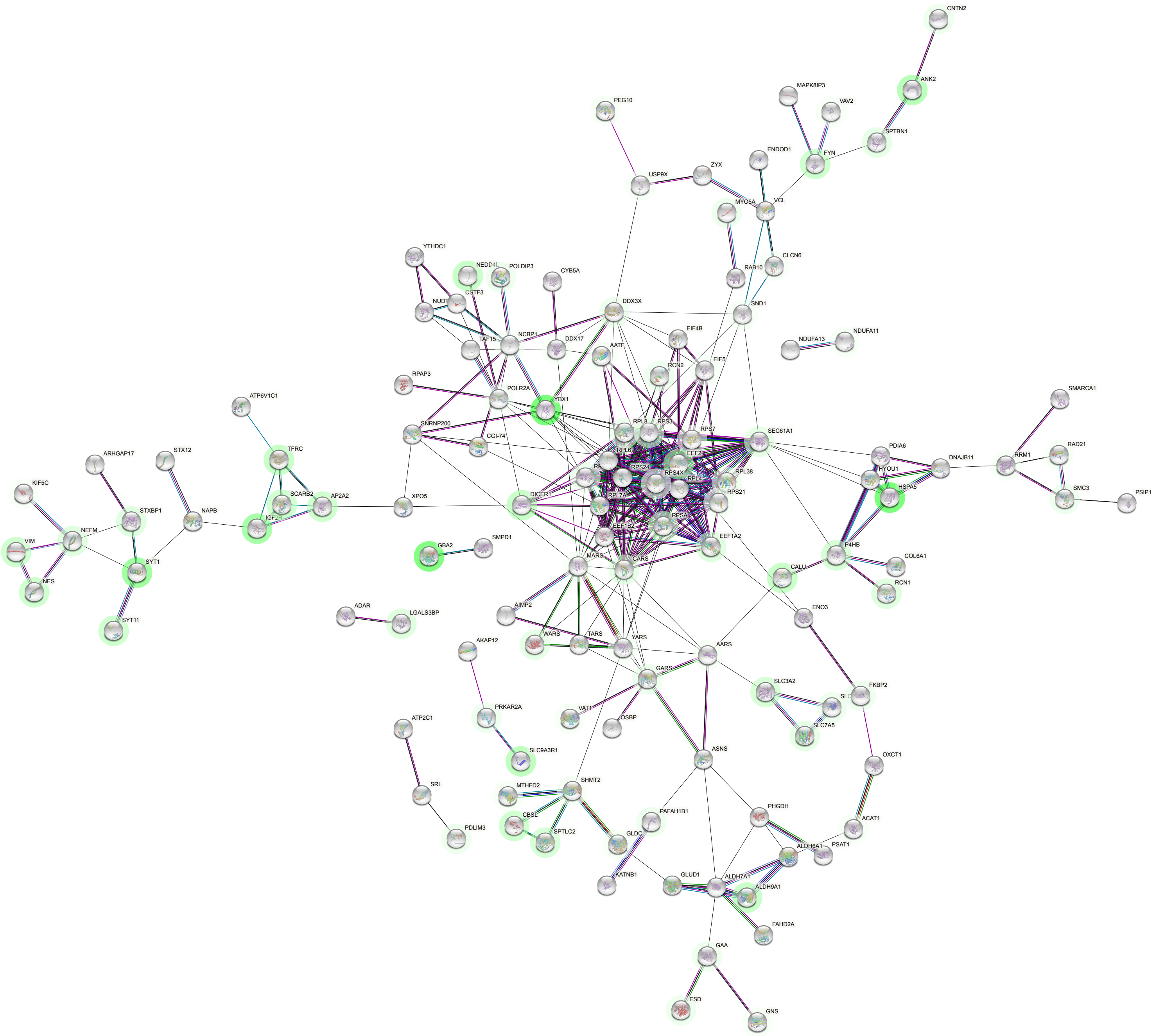

Figure 5. String-DB and clustering analyses of the top 200 genes/proteins. The interaction network was clustered using the MCL clustering algorithm from the String-DB website. The edges between the clusters are removed for visual aid.

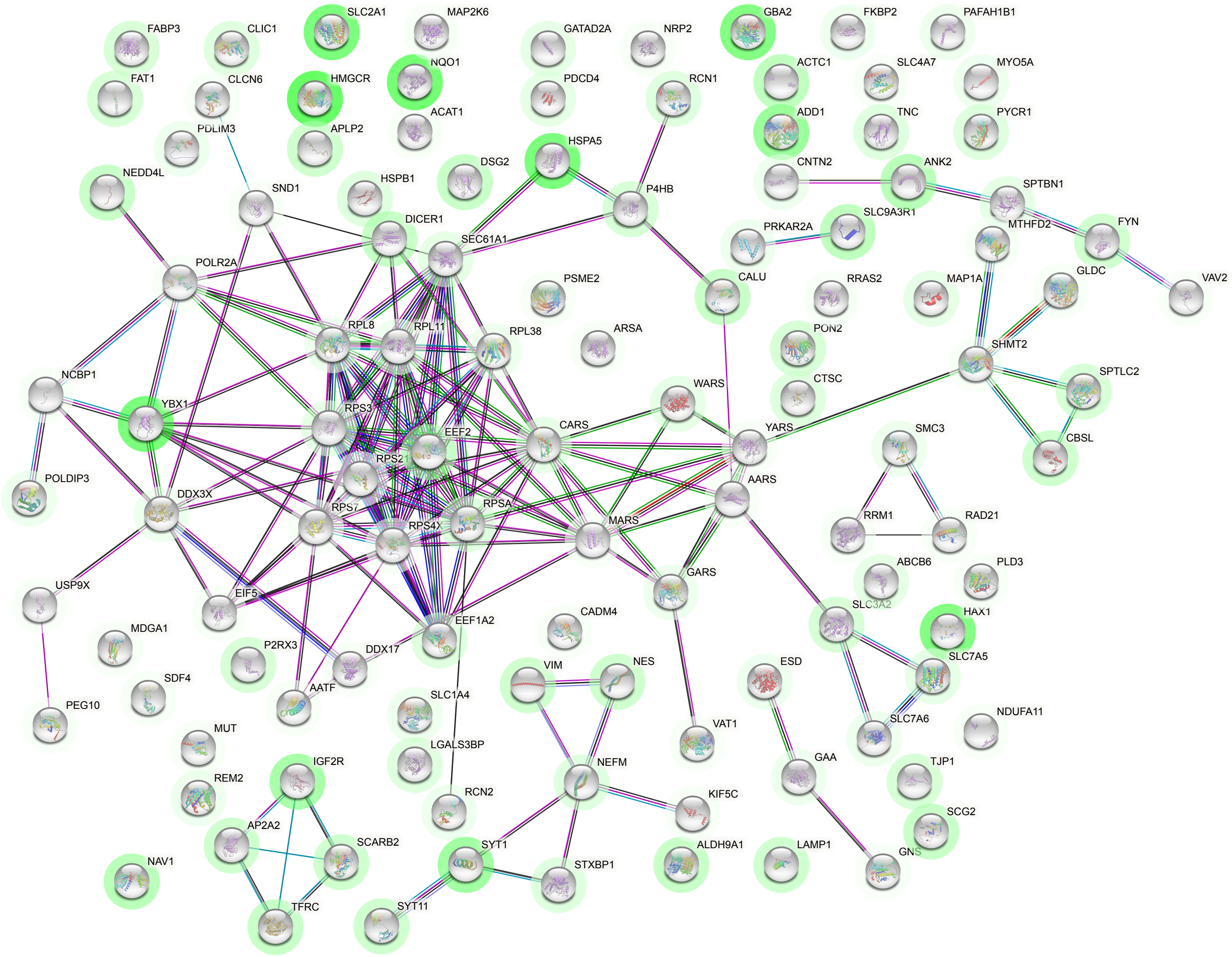

Figure 6. String-DB and clustering analyses of the top 200 genes/proteins. An interaction network is shown for a druggable subset of the top 200 genes/proteins, consisting of 116 genes that were (indirectly) targeted by an FDA-approved drug compound.

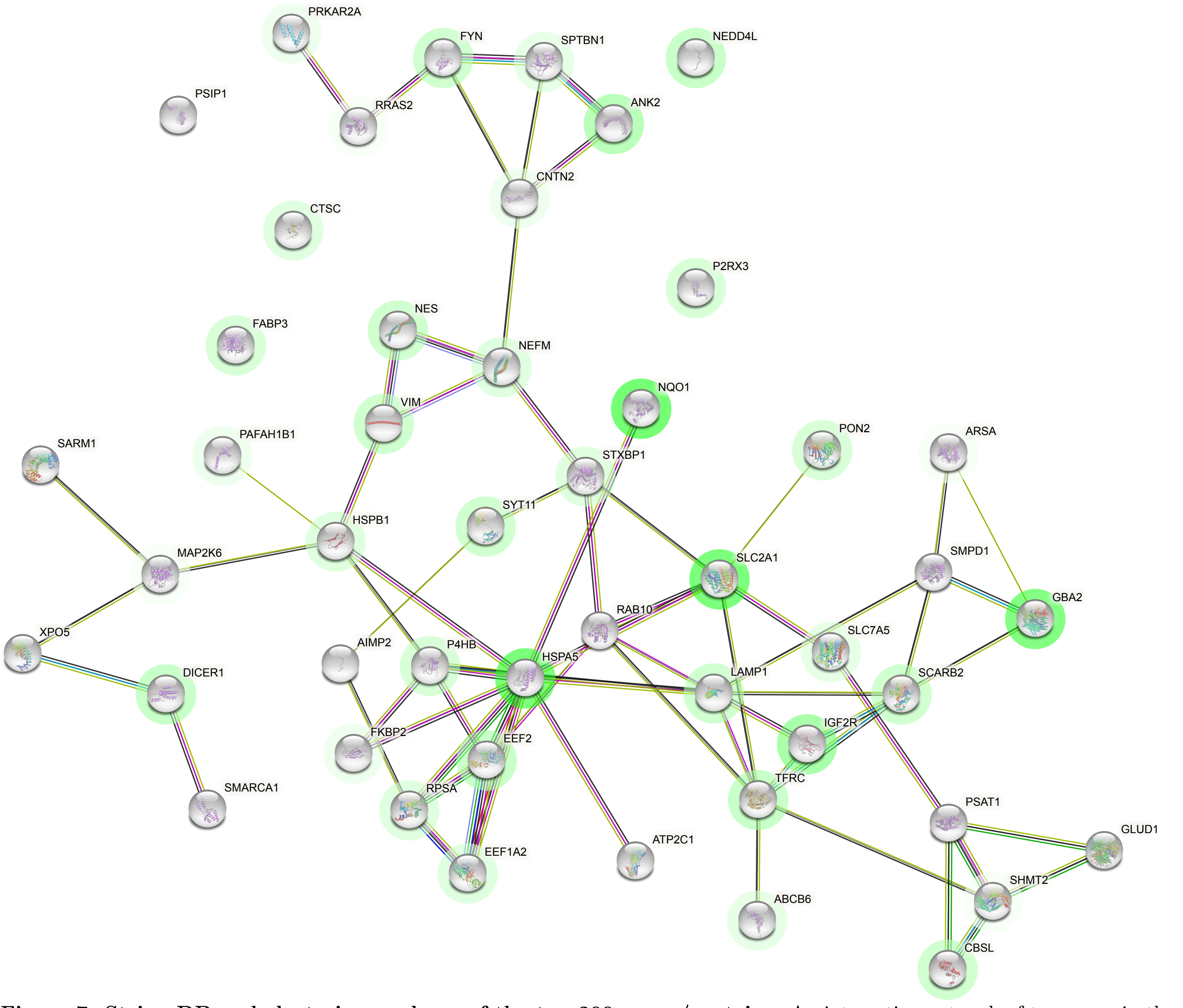

Figure 7. String-DB and clustering analyses of the top 200 genes/proteins. An interaction network of top genes in the "Parkinson's disease" DisGeNet term was constructed using String-DB. Text mining as evidence was included here.

References

1. el Bouhaddani S, Uh HW, Jongbloed G, Houwing-Duistermaat J. Statistical integration of heterogeneous omics data: Probabilistic two-way partial least squares (PO2PLS). J R Stat Soc Ser C (Applied Stat. 2022;doi:10.1111/rssc.12583.

2. Mardia KV, Kent JT, Bibby JM. Multivariate analysis. Academic Press; 1979.
