# Supplementary material for: Statistical integration of multi-omics and drug screening data from cell lines
Source: PLoS Comput Biol. 2024 Jan 31;20(1):e1011809. doi: 10.1371/journal.pcbi.1011809 (PMC10878536; doi:10.1371/journal.pcbi.1011809)
Supplement: Attachment — Submitted filename: ResponseReviewers3.docx [file pcbi.1011809.s002.docx]

In addition to the comments by the authors, please extend the introduction and cite other methods that predict drug effects from cell lines. Position your own previous work and the new work with respect to other studies. Provide guidance for the readers, in how far the current problem cannot be addressed by other methods. Please also include a baseline comparison as suggested by one of the reviewers.

Thank you for the comments. Our method addresses multi-omics data integration augmented with drug screening information in the case of non-overlapping samples. We extended the introduction and positioned our previous and new work with respect to others. Specifically, we revised the introduction to include literature regarding multi-omics integration with non-overlapping samples (p2-3, lines 18-36) and regarding incorporating drug screening data into multi-omics analyses (p3, lines 37-42).

Note that we did not use the multi-omics data to predict drug effects. Instead, our aim is to include the results of drug screen analysis by integrating it with the omics results using information on drug target genes and functional neighboring genes and intersecting this with our omics results. We changed the wording in the introduction to reflect this (p2, lines 5-7).

We clarified that current methods cannot jointly model omics data while allowing for heterogeneity (p2-3, lines 28-36). We further included comparative analyses with several multi- and single-omics approaches (Results: p10; Discussion: p12, lines 262-276; Materials & Methods: p17).

We revised the manuscript and online Supplement according to the reviewers’ comments. Detailed replies can be found below. All changes in the main text are marked in the pdf with a blue color.

Reviewer's Responses to Questions

Reviewer #1: The authors propose a computational pipeline to jointly analyze unpaired multi-omics data from cell lines. As a main part of the workflow, the authors introduce POPLS-DA, a probabilistic orthogonal partial least squares approach for discriminative analysis. For the main results, POPLS-DA was applied to a multi-omics dataset to identify a subset of relevant genes that help discriminate affected from non-affected (aSyn vs control) samples. Subsequently, the authors perform a gene set enrichment analysis on the signature of 200 genes, thereby uncovering significant gene sets / pathways of biological relevance for the dataset at hand. This review focuses mostly on the proposed POPLS-DA method, as it comprises the main source of novelty for the submission.

Major:
My main concern with the paper is the lack of comparison with other baselines. Indeed, the authors suggest that existing approaches assume paired samples, while their proposed method does not. In that case, one may come up with a simple baseline such as analyzing the data modalities independently using existing and widely accepted methods such as tree-based approaches that provide means to quantify the feature/gene importance, and combining the results in a post-hoc manner.

The authors also claim that the results shown in the paper can only be obtained when jointly analyzing the data, and not in a single-omic analysis. However, no evidence is provided to back up this claim.

Thank you for your comments. We did additional comparative analyses including both single- and multi-omics approaches. The conclusion of these additional analyses is that our proposed method was found to perform better than the alternatives.

We added the comparative analyses in the main text on page 10 under subsection “Comparison of POPLS-DA with other omics integration approaches” and on page 17 under paragraph “Comparing POPLS-DA with competing multi- and single-omic approaches”. We also added a paragraph in the discussion regarding the results on page 12, lines 262-276, and a sentence to the abstract and conclusion.

The results of this study is summarized as follows: Besides POPLS-DA, we applied MINT (multi-omics, based on sPLS-DA), LASSO (single-omics, multivariate), and t-tests (univariate) to the transcriptomic and proteomic data. We evaluated interpretability of the selected genes by performing an enrichment test for the DisGeNet Parkinson’s disease and MSA (PD+MSA) disease terms. The top 200 POPLS-DA genes were the most enriched, followed by a t-test on proteomics data and MINT. The other approaches didn’t yield any significant enrichment. Also, intersecting single-omics results did not yield any significant PD+MSA enrichment. We further used permutations to assess predictive ability of each approach under random group labeling. All methods, except LASSO, separated the groups in at most 4 of 400 times. LASSO however achieved perfect accuracy in every randomly permuted dataset. To conclude, it appeared that the POPLS-DA selected genes/proteins were interpretable with respect to PD+MSA and did not suffer from overfitting.

Additionally, we also applied xgboost, using caret’s train and varImp functions, to select the most relevant genes and proteins. However, these approaches showed unstable results and performed worse than LASSO. The PD+MSA enrichment was not significant, while the training accuracy was lower than one. We think that the small sample size and large number of features hampered the algorithm to find a robust ensemble of trees. Therefore, we decided to report the results of penalized linear regression (LASSO) and not report on tree-based analyses in the manuscript.

Minor:
- Figure 1 has poor quality and it is quite difficult to read the text parts.
- In the supplementary material, the table numbering need to be fixed as it currently shows [??] in the reference.
- There are some typos across the manuscript, e.g. page 2: “…the drug screening data to highlight(s) which part…”

We increased the resolution of Figure 1, which is now Fig. 4 (page 15) (see below our response to Reviewer 2, “minor issues”). We further corrected the table numbering in the Supplement and several spelling and grammar mistakes.

Reviewer #2: Summary
In their manuscript “Statistical integration of multi-omics and drug screening data from cell lines” el Bouhaddani and colleagues propose a workflow for multi-omics and drug screening data integration and apply this workflow to study synucleinopathies using transcriptomics, proteomics, and drug screening data on LUHMES cell lines (“cases” samples, overexpressing
-synuclein and “control” samples, overexpressing GFP). In particular the goal of the study is to find genes that distinguish cases and controls and at the same time are druggable.

Multi-modal integration is a common problem in computational biology. Authors developed an extension of PLS-DA method for multi-omics integration that works on non-overlapping samples. Also they developed a workflow for co-analyzing protein-protein interactions between identified genes (that distinguish cases and controls) and drug target information for the most protective drugs (identified in the drug screen). Overall the paper presents an interesting technical approach, but in applying this approach to the biological data in question there are a number of major issues that should be addressed.

Major issues
1. The authors integrate transcriptomics dataset with 15660 transcripts and proteomics dataset with 2577 proteins and in the combined set they get 2292 genes. It means only less than 15% of genes profiled with transcriptomics were used in the analysis. So, if one would use the full transcriptomics dataset (e.g. w/o integration with proteomics) the set of genes that would be identified by a PLS-DA model would be different and also all the downstream findings with respect to drugs - targets analysis. Limiting analysis only to the genes in overlap between two omics sets biases/limits biological findings.

Thank you for the comments. We applied POPLS-DA to only transcriptomics, using the non-filtered 15k transcripts. This approach did not yield any significant enrichment of the DisGeNet Parkinson’s disease and MSA (PD+MSA) gene sets: 16 of 200 genes were in the PD+MSA gene set (FDR p-value was 1.00). These results are not reported. Note that POPLS-DA on single transcriptomics data based on the subset of 2292 genes did provide significant results. These are included in the manuscript (Table 4) and as part of a larger comparative study, see below our response to point 2.

Further, we agree that, whenever it’s possible, one should include all transcripts. However, in our case, the study design with non-overlapping samples hampers an integrative analysis with all 15k genes. See also our response to the additional point below.

One additional point here. Since all samples in the analysis belong to the LUHMES cell line I wonder whether the transcriptomics and proteomics samples can be actually considered as the same i.e. “overlapping” samples. If such consideration is possible one could also perform a “vertical” integration that would preserve all the information in both datasets.

In this study, each sample is a biological replicate derived from a LUHMES cell. Since the transcriptomic and proteomic measurements are obtained from different biological replicates (with different sample sizes), it is impossible to link these data through common sample IDs. Therefore, we developed an approach linking the omics through common genes, taking into account the heterogeneity across omics. We clarified this context in the introduction (p2-3, lines 28-36).

2. The transcriptomics dataset consists of 6 samples, while the proteomics has 18 samples. It’s unclear how well the POPLS-DA deals with cases when the sizes of integrated sets are imbalanced. Would be interesting to see whether the top 200 genes identified by PLS-DA model applied to just proteomics data are different from the top 200 genes obtained in the paper from the integrated dataset.

The sample size was chosen according to the signal-to-noise ratio (estimated in ‘test’ runs in the lab); there, it appeared that proteomic data were more noisy than transcriptomic data. Therefore, more biological replicates were obtained for the proteomic data in the final dataset. We included this text in the discussion (p12, lines 271-276).

We have investigated the implications of the difference in sample size. We applied POPLS-DA to only the proteomics (and transcriptomics) data individually, yielding 42 (resp. 41) out of 200 genes in the PD+MSA gene set (FDR p-value 8.85e-3 resp. 2.15e-2). The overlap of these 42 genes with the original 48 POPLS-DA selected genes was 29 (resp. 19). It appeared that a joint analysis including transcriptomics data yielded more significant enrichment than a single-omics approach based on proteomics data.

These results are included as a part of a larger comparative study, which is added to the main text on page 10 under subsection “Comparison of POPLS-DA with other omics integration approaches” and on page 17 under paragraph “Comparing POPLS-DA with competing multi- and single-omic approaches”. We also added a discussion regarding the results on page 12, lines 262-276. See also our response to Reviewer 1 for a summary of the comparative analyses.

In an additional analysis, we reduced the proteomics sample size by selecting 3 proteomics cases and 3 proteomics controls without replacement to equal the transcriptomics sample size. Across the 84 possible combinations, the median number of POPLS-DA genes in the PD+MSA gene set was 42 (IQR: 38 – 45) out of 200. It appeared that, even with a reduced sample size, the PD+MSA enrichment was still significant. Overall, the POPLS-DA results appeared to be robust against the sample size imbalance in our data. These results are not reported in the manuscript.

3. Page 13, line 311:
“The correlation of the transcripts with the -synuclein overexpressing cells versus the control cells was taken as a reference; if this correlation was negative, the corresponding protein feature was multiplied by minus one.”
This sentence should be re-formulated so it’s clear for the reader what is correlated with what. Also it’s unclear why this per gene correlation is calculated, and what is the reason for the subsequent transformation of protein features.

We reformulated to “We determined the sign of the t-statistic of each gene and protein separately with respect to the case-control grouping. When the sign of the corresponding protein's t-statistic differed from that of the gene, the measurements for that protein were multiplied by minus one. This adjustment ensures that the difference in means between cases and controls had a consistent sign across all omics data.” (p14, lines 366-371).

Minor issues
Fig. 1: the resolution is very low, it’s difficult to read.

Fig. 2, right: although it’s clear that score of 1 means “case” and score of -1 means control, perhaps it should be clarified in the legend or in the main text.

Fig. 3: panel names are missing on the figure. Also bigger font for gene names would make this figure better readable.

Fig. 4: this is a really good and important figure, it could be moved to the front of the paper so the reader understands the whole workflow right from the beginning (I would also show the number of transcriptomics and proteomics samples in the panel (1)). And the current Fig. 1 could be moved to the materials and methods or to the supplement.

**Fig. 1**: (which is now Fig. 4, p15) we increased the resolution.

**Fig. 2**: we added a sentence in the caption: “A positive resp. negative prediction on the *y*-axis corresponds to a case resp. control” (p6).

**Fig. 3**: We added panel names to the figure (p7). Since we found it difficult to increase the fonts for the gene names without introducing overlay issues, we included enlarged versions of the four figures in the Supplementary materials and referred to it in the main text (p4, line 112-113) and caption.

**Fig. 4**: We moved it up to become Fig 1 (p5), referenced in p4, line 83; the ‘old’ Fig. 1 is now Fig. 4.

Supplementary materials, page 4, line 103: “??” symbols instead of Table numbers.

We fixed the Table referencing in the Supplementary materials.

It would be good to have a visual summary / illustration for POPLS-DA method in addition to “mathematical model” and “interpretation” sections in the supplement.

We included a visual illustration of the construction of the POPLS-DA components in terms of predicting the outcome in the Supplementary materials Fig. 1 (suppl page 4).
