# Supplementary material for: Statistical integration of multi-omics and drug screening data from cell lines
Source: PLoS Comput Biol. 2024 Jan 31;20(1):e1011809. doi: 10.1371/journal.pcbi.1011809 (PMC10878536; doi:10.1371/journal.pcbi.1011809)
Supplement: Attachment — Submitted filename: ResponseReviewers_R3.docx [file pcbi.1011809.s003.docx]

Reviewer #2: Authors addressed my comments in the revision, however I've noticed a couple of other statements in the text that would be good to clarify. Also I have questions to the authors's response to my original point number 3.

Line 78: “This approach highlighted genes in the network that are associated with α-synuclein overexpression and targeted by validated drugs, and therefore are potential targets for a novel therapy for synucleinopathies”
If these discovered genes are already targeted by existing drugs why is there a need for a novel therapy targeting the same genes? Conversely if the goal is to discover new potential targets for new therapies why is it important to look at the overlap of genes discovered by multi-omics analysis and genes that are targets of existing protective drugs (and not just to look at all genes discovered by multi-omics analysis).

Thank you for your comments. The goal of the study was to study whether integrating multi-omics and validated drugs can lead to existing drugs being repurposed to Parkinson’s disease (PD) and multiple system atrophy (MSA). These existing drugs are FDA-approved but used for a different indication. Our workflow can help pinpoint drugs that are potentially effective against PD and MSA, as well as highlight targetable genes and proteins underlying the diseases. Novel drug discovery is beyond the scope of this work. We rephrased a sentence in the introduction (p3, l 53-54) to better reflect our goal.

Line 320: “Our novel omics integration POPLS-DA identified a set of 200 relevant genes/proteins that discriminated between samples overexpressing α-synuclein and controls, as well as their drug targets”
It’s unclear to which part of the sentence “as well as their drug targets” belongs. Also, whose drug targets? Drugs can have drug targets, genes/proteins can *be* drug targets but they cannot *have* drug targets.

We changed the last part of the sentence to “as well as validated drugs targeting these genes/proteins” (p13, l 327).

Line 366: “We determined the sign of the t-statistic of each gene and protein separately with respect to the case-control grouping. When the sign of the corresponding protein's t-statistic differed from that of the gene, the measurements for that protein were multiplied by minus one. This adjustment ensures that the difference in means between cases and controls had a consistent sign across all omics data.”

1) Since you are talking about expression / transcriptomics data instead of “gene” you should use “mRNA” or “gene expression level”.
2) It’s unclear to me why the difference in means between cases and controls (for a gene) must have the same sign in transcriptomics and in proteomics data.
3) I understand that such genes (where difference in means between cases and controls in transcriptomics and proteomics data has the different sign) would not be good predictors for case / control prediction, but I am not sure that it’s a justifiable data transformation – to just multiply by -1 a subset of genes in proteomics dataset.

1) We changed the wording from “gene” to “mRNA” whenever we mean only the gene expression measurements.

2+3) In the POPLS-DA estimation, since the effect directions are aggregated across both omics data, the assumption is that this direction is the same across omics levels. If the signs are conflicting, the effects are canceled out. To ensure that both proteins and mRNAs with a relevant effect size are captured, the signs are made to agree over the directions. However, aligning the effect direction may lead to misinterpretation of the effect direction: a selected gene ID can represent overexpression of a protein but underexpression of an mRNA. Since POPLS-DA components are linear combinations of the mRNA and proteins, the sign-correction step can be reversed to get a correct interpretation of the direction if desired. In our motivating data analysis, we focused on any modification of protein or mRNA expression and did not go into detail regarding the direction. We included a discussion regarding this assumption in the Discussion (p13, l 285-290).
